# Similarities and Differences in Genome-Wide Expression Data of Six Organisms

**DOI:** 10.1371/journal.pbio.0020009

**Published:** 2003-12-15

**Authors:** Sven Bergmann, Jan Ihmels, Naama Barkai

**Affiliations:** **1**Departments of Molecular Genetics and Physics of Complex Systems, Weizmann Institute of ScienceRehovotIsrael

## Abstract

Comparing genomic properties of different organisms is of fundamental importance in the study of biological and evolutionary principles. Although differences among organisms are often attributed to differential gene expression, genome-wide comparative analysis thus far has been based primarily on genomic sequence information. We present a comparative study of large datasets of expression profiles from six evolutionarily distant organisms: S. cerevisiae, C. elegans, E. coli, A. thaliana, D. melanogaster, and H. sapiens. We use genomic sequence information to connect these data and compare global and modular properties of the transcription programs. Linking genes whose expression profiles are similar, we find that for all organisms the connectivity distribution follows a power-law, highly connected genes tend to be essential and conserved, and the expression program is highly modular. We reveal the modular structure by decomposing each set of expression data into coexpressed modules. Functionally related sets of genes are frequently coexpressed in multiple organisms. Yet their relative importance to the transcription program and their regulatory relationships vary among organisms. Our results demonstrate the potential of combining sequence and expression data for improving functional gene annotation and expanding our understanding of how gene expression and diversity evolved.

## Abstract 2

Comparative analysis of sequence and gene expression data from bacteria, yeast, worms, flies, weeds, and humans hints at the potential to extract biological and evolutionary principles

## Introduction

Microarray experiments are now being used to address a large diversity of biological issues. The large datasets obtained by pooling those experiments together contain a wealth of biological information beyond the insights gained by individual measurements. For example, it was demonstrated that diverse datasets of genome-wide expression profiles can be applied for facilitating functional assignment of uncharacterized ORFs and for identification of *cis*-regulatory elements (Eisen et al. 1998; Kim et al. 2001; Ihmels et al. 2002).

Comparing the genomic sequences of different organisms presents an alternative prominent approach for gene annotation and identification of regulatory elements (Chervitz et al. 1998; Lynch and Conery 2000; Rubin et al. 2000; Yanai and DeLisi 2002; Frazer et al. 2003). Sequenced-based comparative analyses also proved crucial for deciphering evolutionary principles. As evolutionary changes frequently also involve modifications of the gene regulatory program (Carroll 2000; True and Carroll 2002; Wray et al. 2003), integration of expression data into interspecies comparative analyses could potentially provide new insights into the relation between genomic sequence and organismal form and function. So far, however, such an approach has been mostly applied to small numbers of genes (Carroll 2000; True and Carroll 2002; Wray et al. 2003) or has been restricted to variations in the genome-wide expression profiles during the development of closely related species (Rifkin et al. 2003). With the accumulation of large-scale expression data for a number of diverse species, the time may be ripe for a macro-evolutionary comparison of gene expression.

Expression data differ from sequence data in two main aspects, which make their integration into comparative analysis challenging. First, unlike sequence information, which is direct and accurate, expression profiles provide only indirect and noisy information about the regulatory relationships between genes. Second, while the genomic sequence is essentially complete, expression profiles only cover a subset of all possible cellular conditions and thus provide only partial information about the underlying regulatory program. Moreover, this subset is typically very different for each organism, reflecting distinct physiologies as well as different research foci. One way to circumvent this problem is to restrict the data to a small subset of similar conditions, such as timepoints along the cell cycle (Alter et al. 2003). Such an approach, however, drastically reduces the size of the dataset and limits the scope of comparison.

Here, we present a comparative analysis of large sets of expression data from six evolutionarily distant organisms (Table 1). We integrate the expression data with genomic sequence information to address three biological issues. First, we verify that coexpression is often conserved among organisms and propose a method for improving functional gene annotations using this conservation. We provide a Web-based application suitable for this purpose. Second, we compare the regulatory relationships between particular functional groups in the different organisms, giving initial insights into the extent of conservation of the gene regulatory architecture. Interestingly, we find that while functionally related genes are frequently coexpressed in several organisms, their organization and relative contribution to the overall expression program differ. Finally, we compare global topological properties of the transcription networks derived from the expression data, using a graph theoretical approach. This analysis reveals that despite the differences in the regulation of individual gene groups, the expression data of all organisms share large-scale properties.

**Table 1:**
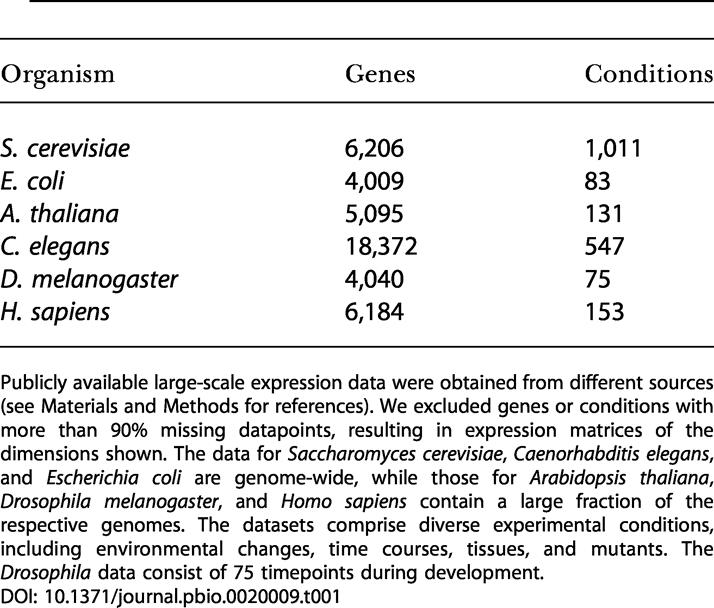
Large-Scale Expression Data Used in This Study

Publicly available large-scale expression data were obtained from different sources (see Materials and Methods for references). We excluded genes or conditions with more than 90% missing datapoints, resulting in expression matrices of the dimensions shown. The data for Saccharomyces cerevisiae, Caenorhabditis elegans, and Escherichia coli are genome-wide, while those for Arabidopsis thaliana, Drosophila melanogaster, and Homo sapiens contain a large fraction of the respective genomes. The datasets comprise diverse experimental conditions, including environmental changes, time courses, tissues, and mutants. The *Drosophila* data consist of 75 timepoints during development

## Results and Discussion

### Combining Sequence and Expression Data for Improving Functional Gene Annotations

With the rapid increase in the number of sequenced genomes, assigning function to novel ORFs has become a major computational challenge. Functional links are often imputed based on sequence similarity with genes of known functions. Despite the large success of this approach, it has several well-recognized limitations. Foremost, an ORF can have several close homologues, some of which may be related to different functions. Furthermore, the sequence of an ORF may have diverged beyond recognition although the gene maintained its function.

Gene expression analysis can provide functional links for new ORFs based on their coexpression with known genes. However, in this case, only links between genes of the same organism can be established. Moreover, owing to biological interference and the noise in the expression data, the inferred coexpression could be accidental and may not necessarily reflect similar function.

**Figure 1:**
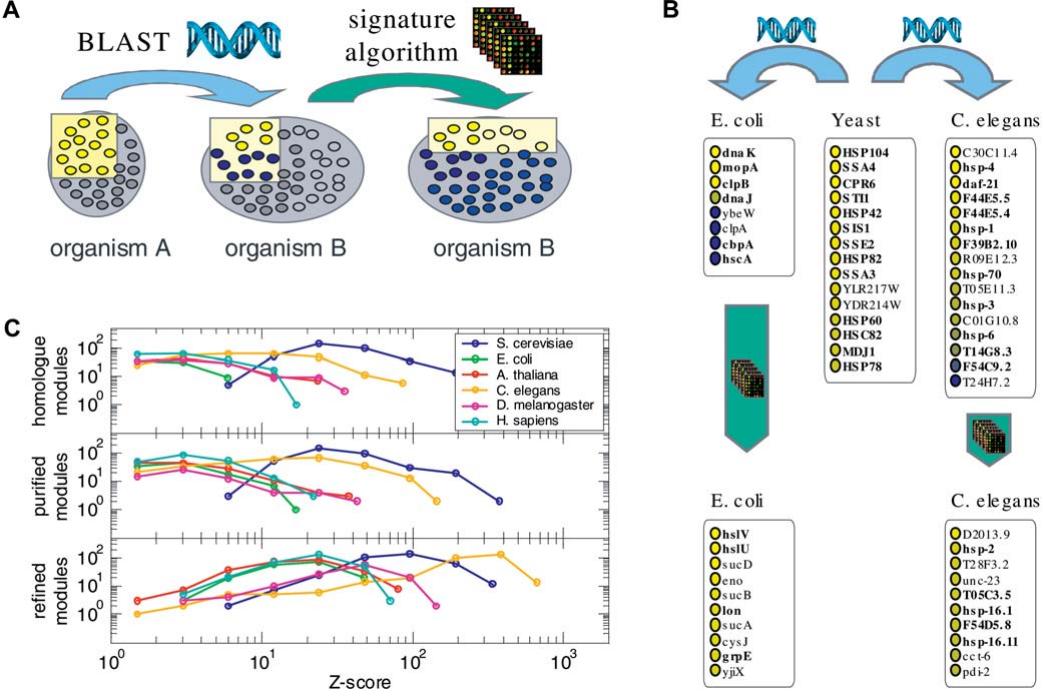
Using Expression Data to Identify and Refine Sequence-Based Functional Assignments (A) Starting from a set of coexpressed genes (yellow dots in left box) associated with a particular function in organism A, we first identify the homologues in organism B using BLAST (middle box). Only some of these homologues are coexpressed while others are not (blue dots). The signature algorithm selects this coexpressed subset and adds further genes (light yellow) that were not identified based on sequence, but share similar expression profiles (right box). (B) The 15 coexpressed genes associated with heat shock in yeast (center) have eight homologues in E. coli (left) and 14 in C. elegans (right). Among the ten genes whose expression profiles are the most similar to these homologues (bottom), many are known to be associated with heat-shock response (boldface). (C) For each of the six organisms, the distribution of the *Z*-scores for the average gene–gene correlation of all the “homologue modules” (see Materials and Methods) obtained from the yeast modules is shown (top). Rejecting the homologues that are not coexpressed gives rise to the “purified modules,” whose *Z*-scores generally are larger (except for the yeast modules, which contain only coexpressed genes from the beginning). Adding further coexpressed genes yields the “refined modules,” which have significantly larger *Z*-scores (bottom).

Combining expression and sequence data may help to overcome the abovementioned limitations. Specifically, homologous genes whose function has been preserved are expected to be coregulated with genes related to that function. Conserved coexpression could thus distinguish them from homologues whose function diverged. This can be done, for example, by focusing on a group of functionally related genes in a characterized genome, identifying simultaneously all the respective homologues in a second genome, and then examining which of the homologues are indeed coexpressed (Figure 1A). Importantly, restricting the search for coexpressed genes to a limited set of candidates provides an effective mean to overcome the noise in the expression data (Ihmels et al. 2002).

#### Conserved coregulation of functionally related genes

To explore systematically the utility of this approach, we first examined to what extent coexpression is conserved among different organisms. We performed a statistical analysis comparing the pairwise correlations between genes in one organism to the correlations between their respective homologues. Indeed, a significant fraction of such correlations were similar (see Figure S8). The strongest conservation of coexpression was found between pairs of genes associated with particular cellular processes, such as core metabolic functions or central complexes (e.g., ribosome and proteasome) (lists of gene pairs with conserved coexpression are available at http://barkai-serv.weizmann.ac.il/ComparativeAnalysis).

Next, we examined whether coexpression is conserved among groups of genes that are associated with the same cellular function. To this end, we used as a benchmark coexpressed groups of genes (termed transcription modules; see Materials and Methods for a precise definition) that we extracted from the Saccharomyces cerevisiae expression data (Ihmels et al. 2002; J. Ihmels, unpublished data). (The yeast data are the most comprehensive and best annotated, resulting in a large number of transcription modules that can be associated with a specific cellular function.) For each yeast module, we constructed five “homologue modules,” which contain the respective S. cerevisiae homologues in the other organisms, and measured the correlation between the genes of these homologue modules. The average correlation between the genes of the homologue modules was indeed statistically significant (see the top panel of Figure 1C), indicating that coexpression of functionally linked genes is often conserved among organisms.

#### Coexpression can be used for refining homologue modules

Examining the pairwise correlations themselves, however, revealed that usually only a fraction of the genes are correlated with each other (see Figure S9). Such lack of correlation probably reflects the inadequacy of defining function solely based on homology. To search for a coexpressed subset within each homologue module, we applied the signature algorithm we proposed recently (Ihmels et al. 2002). The algorithm identifies those homologues that are coexpressed under a subset of the experimental conditions. Furthermore, it reveals additional genes that are not homologous with any of the original genes, but display a similar expression pattern under those conditions (see Materials and Methods). Studying the output of the algorithm, we found that the rejected homologues are usually not associated with the original function, while many of the added genes are. For example, from the 15 coexpressed yeast genes involved in heat-shock response, we identified eight homologues in Escherichia coli and 16 in Caenorhabditis elegans. While only some of these homologues are highly coexpressed, they are sufficient to retrieve additional genes known to be involved in heat shock (Figure 1B; see Figure S10 for other modules).

A statistical analysis using all yeast modules revealed that many homologue modules are significantly coexpressed. The extent of coregulation increases drastically upon removing uncorrelated homologues and adding related genes (Figure 1C). We note that in some cases such a “purified module” may contain two or more distinct coexpressed groups. Such substructures are identified by clustering all pairwise gene correlations (see Data S3). We conclude that sequence-based functional annotation can be significantly improved through the integration of expression data. We provide an interactive tool for this purpose on our Web site at http://barkai-serv.weizmann.ac.il/ComparativeAnalysis (see also Figure S7). We note that while this paper was in review, the possibility of enhancing functional assignment based on the conservation of coexpression was reported independently by Stuart et al. (2003).

### Higher-Order Regulatory Structures

#### Regulatory relations between functional groups vary among organisms

**Figure 2:**
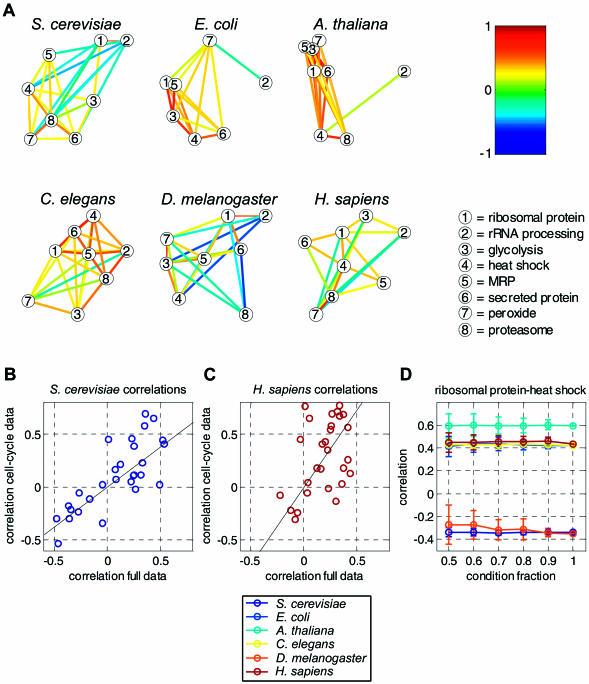
Regulatory Relations between Modules A selection of eight transcription modules whose function is known in yeast was used to generate the corresponding (refined) homologue modules in the other five organisms. Each module is associated with a “condition profile” generated by the signature algorithm based on the expression data. (A) Correlations between these profiles were calculated for all pairs of modules in each organism. Note that for E. coli there is no proteasome and that the mitochondrial ribosomal proteins (MRPs) correspond to ribosomal genes. Modules are represented by circles (legend). Significantly correlated or significantly anticorrelated modules are connected by colored lines indicating their correlation (color bar). Positively correlated modules are placed close to each other, while a large distance reflects anticorrelation. See Figure S11 for a numerical tabulation of all pairwise correlations. (B and C) Correlations between pairs of modules according to the cell-cycle data as a function their correlation in the full data. Each circle corresponds to a pair of S. cerevisiae modules (B) or human modules (C). (D) To check the sensitivity of our results with respect to the size of the dataset, we reevaluated the correlations between the sets of conditions for randomly selected subsets of the data. Shown are the mean and standard deviation of the correlation coefficient between the heat-shock and protein-synthesis modules as a function of the fraction of removed conditions (see Figures S4 and S5 for correlations between other module pairs).

The observation that groups of functionally related genes are often coexpressed in multiple organisms prompted us to ask whether also the higher-order regulatory relationships between these groups have been conserved (see Materials and Methods). To address this question, we focused on eight representative yeast modules related to cellular core processes. Several of the regulatory relations among the homologues of these modules have been conserved (Figure 2A). For example, in all organisms the modules associated with protein synthesis and protein secretion are positively correlated, while the rRNA synthesis and the peroxide modules are anticorrelated. Interestingly, however, most of the relations between modules differ among organisms. In particular, one of the prominent features of the yeast transcription program, namely the strong anticorrelation between heat-shock and protein-synthesis modules (Ihmels et al. 2002), was observed only in the yeast and *Drosophila* data. In contrast, those two modules displayed a significant positive correlation in the expression data of all other organisms. We note that both types of regulation are consistent with the role of heat-shock proteins as chaperones; it appears that in yeast their primary role is to assist in protein folding during stress conditions (when ribosomal protein genes are repressed), while in the other organisms they may be required to accelerate folding during cell growth.

In order to test whether the variations in the regulatory relations among functional groups in different organisms are due to the use of unrelated sets of experimental conditions, we restricted both the human and the yeast expression data to the cell cycle experiments. We found that the correlations between modules did not change qualitatively due to this restriction (Figure 2B and 2C). We also examined the sensitivity of our results to the number of conditions used (see Materials and Methods). Removal of up to 50% of all conditions did not considerably change the gene content of most refined modules (see Data S2). Importantly, this analysis also revealed that the correlations between modules are insensitive to the subset of conditions used (Figure 2D; see also Figure S2). Note, for example, that for the largest datasets (yeast and C. elegans), the standard deviations of the correlation coefficients do not exceed 0.1, even when removing half of the expression profiles. Taken together, these results indicate that, despite the sparseness of the data, our findings reflect real properties of the expression networks and not the specific subset of experimental conditions used.

#### Global decomposition of the expression data of different organisms

**Figure 3:**
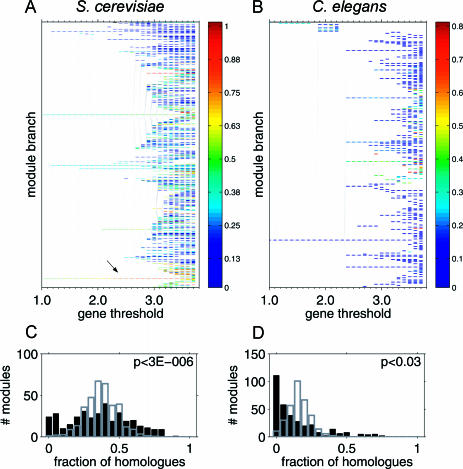
Properties of Transcription Modules (A and B) Module trees summarize the transcription modules identified by the ISA at different resolutions. Branches represent modules (rectangles) that remain fixed points over a range of thresholds. Fixed points that emerge at a higher threshold converge into an existing module when iterated at a lower threshold (thin transversal lines). Modules are colored according to the fraction of homologues they possess in the other organism (see the color bar). Among the yeast modules, those associated with protein synthesis (arrow) have the largest fraction of worm homologues. Searchable trees for all six organisms are available at http://barkai-serv.weizmann.ac.il/ComparativeAnalysis. (C) Histogram for the number of yeast modules with a given fraction of genes possessing a homologue in C. elegans (black bars). The distribution indicates that a significant number of modules have either much less or much more homologues than expected; indicated *p*-value were computed according to Kolmogorov–Smirnov test against control distribution (gray) generated from random sets of modules preserving their size. (D) Same as in (C) for C. elegans modules considering yeast homologues (see Figure S12 for other organisms).

To compare the higher-order regulatory structures more systematically, we decomposed the expression data of each organism into a set of transcription modules using the iterative signature algorithm (ISA) we proposed recently (Bergmann et al. 2003; J. Ihmels, unpublished data). A transcription module consists of coexpressed genes and the conditions that induce their coregulation. Importantly, the stringency of coregulation is determined by a threshold parameter, which allows for a modular decomposition at different resolutions. At low resolution, a few relatively large transcription modules are identified. At higher resolution, the data are usually decomposed into a large number of modules, which contain fewer but more tightly regulated genes. We visualize the modular decomposition by a module tree (Figure 3A and 3B). Highly similar modules, identified at adjacent thresholds, are connected by lines and define the branches of the tree. In contrast to the common dendrograms used to summarize the results of hierarchical clustering, here distinct branches may share common genes, and when two branches merge, the resulting branch is not necessarily their union.

#### Modular architectures of the transcription programs are distinct

Modular architectures, as reflected by the structure of the associated module trees, vary greatly among organisms. Differences were observed in the total number of modules, the threshold ranges over which modules are stable, and the overall hierarchical organizations. For example, in yeast the data were composed into just five transcription modules at low resolution, which remained stable for a wide range of thresholds (Figure 3A). As we reported previously (Bergmann et al. 2003), these modules correspond to the central yeast functions (protein synthesis, stress, amino-acid biosynthesis, cell cycle, and mating). At high resolution, a large number of modules with specific cellular functions were identified. The corresponding module tree reveals a clear hierarchy in the transcriptional network, with gradually increasing complexity. In contrast, the C. elegans tree exhibits a sharp transition between a regime dominated by a single branch (from which only few less-stable modules branch off) to a part of the tree that rapidly bifurcates into many branches at higher thresholds (Figure 3B).

Interestingly, the functional groups that dominate the transcription program of each organism are also distinct. For example, in S. cerevisiae and E. coli, genes coding for ribosomal proteins are associated with a central branch that persists over a wide range of thresholds, reflecting the large number of the experimental perturbations that induce the coregulation of these genes. In contrast, although ribosomal proteins are also coregulated in higher organisms, they are associated with short branches that extend only over a small range of thresholds. This suggests that transcriptional regulation of genes involved in protein synthesis plays a major role in the transcription program of unicellular organisms, but a less dominant role in multicellular organisms.

#### Conserved and organism-specific transcription modules

We observed that several functional groups were repeatedly identified as coexpressed in several organisms. This includes modules related to core biological functions such as protein synthesis, rRNA processing, the proteasome, and oxidative phosphorylation. Still, most of the transcription modules were observed in just one organism. In order to distinguish more systematically between generic modules and those that are involved in an organism-specific function, we determined for each module the fraction of genes that possess at least one homologue in a second organism (see Materials and Methods). For S. cerevisiae and C. elegans (the two largest datasets), most modules have either significantly less or significantly more homologues than expected (Figure 3C and 3D). This indicates that while a number of generic modules have been conserved under evolution, each transcriptome also contains more recently evolved modules that are associated with organism-specific functions.

### Comparing Global Features of Gene Expression Networks

#### Power-law connectivity distribution

**Figure 4:**
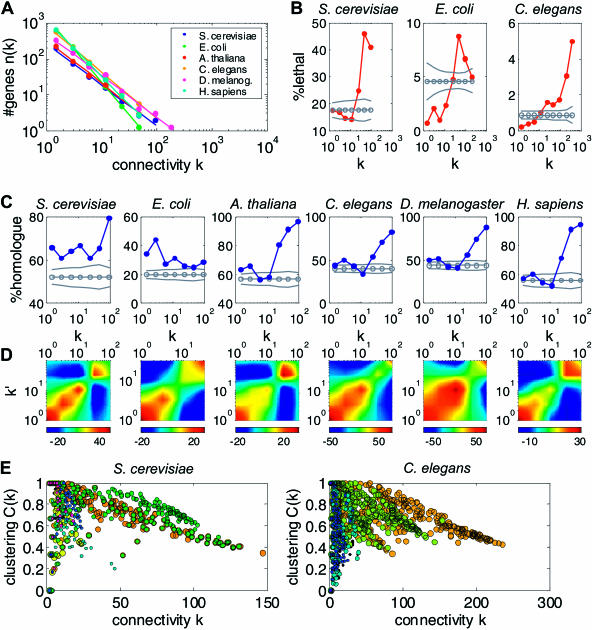
Global Properties of Transcription Networks (A) The number of genes *n(k)* with connectivity *k* is plotted as a function of *k* (see Materials and Methods). For each of the six organisms *n(k)* is distributed as a power-law, *n(k)* ∼ *k* ^−γ^, with similar exponents γ ≈ 1.1–1.8 (see Figure S13). (B) The fraction of lethal genes is shown as a function of *k* for S. cerevisiae, E. coli, and C. elegans. The control (gray line) is obtained from 10,000 random choices for the lethal genes (preserving their total number). The dashed lines indicate standard deviations. (C) The fraction of genes with at least one yeast homologue is shown as a function of *k* for all six organisms. Control (gray) as in (B). (D) *Z*-score quantifying the deviation of the number of connections between genes with connectivities *k* and *k*′ from that expected by randomly rewired networks (see Maslov and Sneppen 2002). Note that connections between genes of similar connectivity are enhanced (red regions), while those between highly and weakly connected genes are suppressed (blue). (E) The clustering coefficient *C* is plotted against *k*. Each dot corresponds to a single gene and is colored according to the transcription module it is associated with (see also Figure 2). Note that genes associated with the same module correspond to a specific band in the *k*–*C* plane. Several genes with high connectivity belong to more than one module (green dots superimposed on orange ones).

We next sought to compare global topological properties of the expression data. To this end, we represented the data by an undirected “expression network,” whose nodes correspond to genes. Two genes are connected by an edge if their expression profiles are sufficiently correlated (see Materials and Methods). We use this mapping to explore the global structure of the expression data using tools of graph theory. A well-established indicator of the network topology is the distribution *n(k)* of the connectivity *k* (the number of edges of a particular gene). We find that for all organisms, the connectivity is distributed as a power-law, *n(k)* ∼ *k* ^−γ^, with similar exponents γ ≈ 1.1–1.8 (see Figure 4A). The expression networks thus belong to the class of scale-free networks, which comprises many real-world networks (Albert and Barabasi 2002). Power-law distributions have been attributed to dynamically evolving networks (Barabasi and Albert 1999) and to systems that are optimized to provide robust performance in uncertain environments (Doyle and Carlson 2000). In the present context, a power-law connectivity distribution indicates that there is no typical size for sets of coexpressed genes and that there is a significant enrichment of highly connected genes as compared to random networks (see also Guelzim et al. 2002; Lee et al. 2002; Shen-Orr et al. 2002).

#### Highly connected genes are often essential and evolutionarily conserved

To see whether higher-order features of the connectivity distribution are also conserved, we calculated the likelihood *P(k, k′)* that two genes of connectivity *k* and *k*′, respectively, are connected with each other (Maslov and Sneppen 2002). In all expression networks, connections between genes with similar connectivity occur much more often than expected, while connections between highly and weakly connected genes are suppressed (Figure 4D). The common topology of the expression networks is thus different from the topology of the yeast protein–protein interaction network, although both exhibit a scale-free connectivity distribution (Maslov and Sneppen 2002).

We next examined whether highly connected genes are involved in central biological functions. In yeast, most of such genes are associated with protein synthesis, in particular rRNA processing. In the other organisms, the functional role of the highly connected genes is different and less coherent (lists of these “hub” genes are available at http://barkai-serv.weizmann.ac.il/ComparativeAnalysis). Interestingly, in the three organisms in which large-scale knockout information is available (see Materials and Methods), the likelihood of a gene to be essential increases with its connectivity (Figure 4B). Similar results were recently reported for the yeast expression and protein–protein interaction networks (Jeong et al. 2001; Farkas et al. 2003). We also observed that the highly connected genes are more likely to have homologues in the other organisms (Figure 4C). This finding is consistent with the framework of dynamically evolving networks, where nodes that were added at an early stage (and may thus correspond to highly conserved genes) are more likely to develop many connections.

#### Expression networks are highly clustered

A further indicator of the network structure is the clustering coefficient *C*, which quantifies the degree of modularity (Watts and Strogatz 1998). For expression networks, *C_g _* measures to what extent the genes connected to a specific gene *g* are also connected with each other (see Materials and Methods). The networks of all organisms exhibit a high modularity with 〈*C_g_*〉 ≈ ½, several orders of magnitude higher than what would be expected for random networks (Albert and Barabasi 2002). We also examined the relation between the clustering coefficient and the connectivity of each gene. For all six organisms, we observed an approximately triangular region in the *k–C* plane where genes clustered into several localized elongated regions (Figure 4E). Within these “bands,” the clustering coefficient decreases monotonically as a function of the connectivity. Recently, a similar monotonic relation was observed in metabolic networks as well as in several nonbiological networks (Ravasz et al. 2002; Ravasz and Barabasi 2003). For random networks and for simple dynamically evolving networks, it was shown that *C* is independent of *k*. However, deterministic models that lead to a hierarchical organization of modularity predict *C* ∼ *k* ^–1^ (Dorogovtsev et al. 2002; Ravasz and Barabasi 2003).

Intriguingly, we found that genes belonging to the same band are often coexpressed and associated with one of the dominating coexpressed units (transcription modules) identified by our modular analysis. The decrease of *C* as a function of *k* may reflect overlap between modules. Genes that are associated with only one module have a connectivity reflecting the size of the module and a large clustering coefficient. In contrast, genes that belong to several modules are correlated with a larger number of genes, but many of these genes are not connected with each other, leading to a smaller clustering coefficient. In support of this, we found that highly connected genes with a small clustering coefficient are often associated with several modules (Figure 4E). Thus, the band-like structures we observed may reflect the combinatorial regulation of gene expression.

## Conclusions

Comparing genomic properties of different organisms is of fundamental importance in the study of biological and evolutionary principles. Although much of the differences among organisms is attributed to different gene expression, comparative analysis thus far has been based primarily on genomic sequence information. The potential of including functional genomic properties in a comparison analysis was demonstrated in recent studies that compared protein–protein interaction networks of different organisms (Matthews et al. 2001; Kelley et al. 2003).

In this paper we presented a comparative analysis of large datasets of expression profiles from six evolutionarily distant organisms. We showed that all expression networks share common topological properties, such as a scale-free connectivity distribution and a high degree of modularity. While these common global properties may reflect universal principles underlying the evolution or robustness of these networks, they do not imply similarity in the details of the regulatory programs. Rather, with a few exceptions, the modular components of each transcription program as well as their higher-order organization appear to vary significantly between organisms and are likely to reflect organism-specific requirements.

Nevertheless, coexpression of functionally linked genes is often conserved among several organisms. Based on this finding, we proposed an efficient method that uses coexpression analysis for improving sequence-based functional annotation. An interactive implementation of this algorithm is available at http://barkai-serv.weizmann.ac.il/ComparativeAnalysis/.

Our analysis was based on the available expression data, which are still sparse for most organisms. It is likely that the modular decompositions we obtained are partial, so additional modules can be identified as more expression data become available. Nevertheless, by analyzing the sensitivity of our results to the number of conditions, we concluded that the composition of the modules themselves is rather robust. Moreover, the higher-order correlations between modules are only slightly affected by the number of conditions.

The absence of a large set of common experimental conditions, however, does limit the scope of the present analysis and reduces the possibility of addressing particular evolutionary issues. It would be interesting, for example, to compare how different organisms respond to a variety of stress conditions, which were found to induce a unified transcription program in S. cerevisiae (Gasch et al. 2000). Similarly, it would be intriguing to examine whether knockouts of homologous genes induce a similar transcriptional response in the different organisms. Comparative studies of gene expression pattern could be largely facilitated by unified datasets, which examine the genome-wide expression profiles of diverse as well as related species, under comparable experimental conditions.

## Materials and Methods

### 

#### Expression data

Preprocessed expression data from E. coli, Arabidopsis thaliana, and Homo sapiens were downloaded from the Stanford Microarray Database (Sherlock et al. 2001) using default parameters and selecting data from all experimenters and categories. For technical reasons (see Data S5), we only used the first 720 experimental conditions for the human dataset or all conditions related to the cell cycle. C. elegans expression data were obtained from Kim et al. (2001) and Drosophila melanogaster data from Arbeitman et al. (2002). The yeast expression data (Gasch et al. 2000; Hughes et al. 2000; Causton et al. 2001) contain more than 1,000 experiments (see http://barkai-serv.weizmann.ac.il/modules/page/references.html for a complete list of references). We excluded genes or conditions with more than 90% missing datapoints, resulting in expression matrices of the dimensions shown in Table 1 (see Data S4 for comment on missing values in the expression data).

#### Sequence data

FASTA files for amino-acid sequences of coding regions were downloaded from the sources detailed in Table 2. We ran the BLASTP 2.2.2 (Altschul et al. 1997) locally in order to determine the sequence similarity among all coding regions. Gene/ORF identifiers were used to link the sequence data with the expression profiles.

**Table 2:**
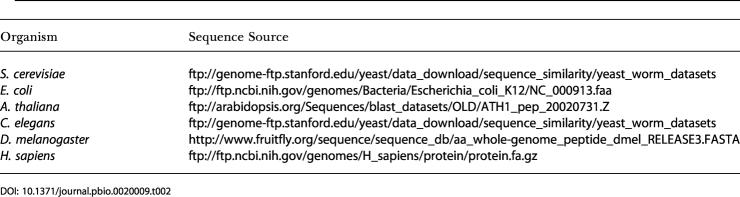
The Sources of the Sequence Data Used in This Study

#### Knockout data

Data for deletion mutants (S. cerevisiae and E. coli) and RNAi experiments (C. elegans) were obtained from the sources indicated in Table 3. Note that the fraction of the genome that was tested for viability varies among the three organisms.

**Table 3:**
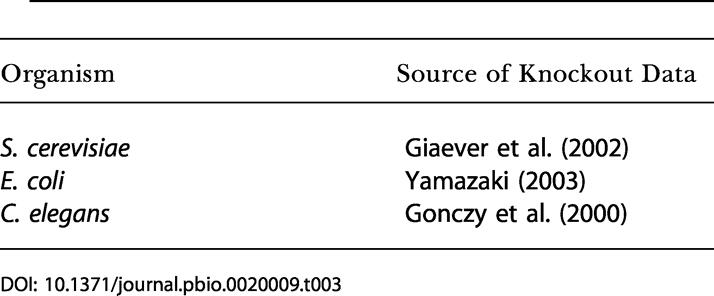
Sources of Knockout Data Used in This Study

#### Module definition

A transcription modules consist of a set of coregulated genes (a subset *G_m_* of the genome *G*) and an associated set of regulating conditions (a subset *C_m_* of all conditions *C*). The defining property of a transcriptional module is self-consistency, which is achieved as follows. First, we assign scores to both genes and conditions that reflect their degree of association with the module. The gene score is the average expression of each gene over the module conditions, weighted by the condition score: 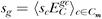. Analogously, the condition score is the weighted average over the module genes, 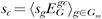. Here, 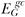 and 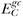 are the log-expression ratio of gene *g* in condition *c* normalized over genes and conditions, respectively, such that 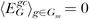, 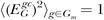 and 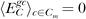, 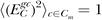. Self-consistency denotes the property that the genes of the module are those genes of the genome that receive the highest scores *s_g_*, while the module conditions are those conditions in the dataset with the highest scores *s_c_*. The ISA identifies transcription modules through iterative refinement of a large number of random gene scores.

#### Module analysis

For the analysis of the fraction of homologues (see Figure 3C and 3D) as well as the average pairwise correlations (see Figure 1C), we used most of the transcription modules identified by the ISA. In order to avoid bias from similar modules identified at adjacent thresholds, we considered only modules with less than 70% similarity to any module identified at a lower threshold. Two sequences were considered homologues if they could be aligned along at least 40% of the shorter sequence by the BLAST algorithm and obtained an E-value smaller than 10^–5^. The precise parameter values have only a minor effect on our results (see Data S1 for detailed statistical analysis). We only considered modules with at least five homologues.

#### Module purification and refinement

A “homologue module” consists of the genes homologous to a transcription module in another organism. We used the signature algorithm to purify and refine these homologue modules (see Ihmels et al. 2002 for details of the algorithm). A “purified module” is the intersection between the homologue module, used as input for the signature algorithm, and the resulting output. It contains only genes that are coexpressed. A “refined module” is obtained by applying the signature algorithm again, this time using the purified module as input. The output consists both of the coexpressed genes and the conditions inducing their coexpression. This twofold application of the signature algorithm usually provides a more accurate determination of the coexpressed genes related to the original transcription module than a single application. In order to also capture weakly coexpressed modules, we used relatively low thresholds (*t_g_* = *t_c_* = 1.5) in the present analysis, but retained only genes whose score is not less than 70% of the most significant gene (Ihmels et al. 2002).

#### Correlations between modules

Both a transcription modules and the refined homologue module derived from it are associated with a set of coregulating experimental conditions (Ihmels et al. 2002). The significance of each condition is characterized by a score *s_c_*. The sets of scores can be used to compute the regulatory relation between two modules of the same organism. We use *C_ij_* = (Σ_c_*s*_*c*_^(*i*)^ ·*s*_*c*_^(*j*)^)/(Σ_c_*s*_*c*_^(*i*)^ ·*s*_*c*_^(*i*)^ ·Σ_c_*s*_*c*_^(*j*)^ ·*s*_*c*_^(*j*)^) ^½^ as the correlation coefficient between two modules with score sets {*s*_*c*_^(*i*)^} and {*s*_*c*_^(*j*)^}, respectively. Note that, unlike for the Pearson correlation, this definition of *C_ij_* does not center the scores.

#### Network analysis

Each expression network can be described by a symmetric adjacency matrix *A_ij_*, whose elements are 1 if the expression of gene *i* and gene *j* are sufficiently similar and 0 otherwise. Similarity was measures by the Pearson correlation coefficient between the expression profiles. Owing to the very different sizes of the respective sets of expression data, we demanded that the average connectivity <*k*> (rather than the minimal correlation) is identical in all expression networks and fixed it to <*k*> = 0.001. Using the top 0.1% of all possible correlations corresponds to a lower limit on the correlation coefficients between 0.63 for S. cerevisae and 0.85 for D. melanogaster. The results are insensitive to the precise threshold value (see Figure S2 for detailed analysis). The connectivity of gene *i* is *k* = Σ_*j*≠*i*_ *A_ij_*. In order to obtain the connectivity distributions *n(k)*, we used logarithmic binning. The edges of the bins were powers of 2, and we counted the number of genes with *k_i_* between two edges and normalized by the bin width. We applied a linear fit to the log values of the bin centers against the normalized counts. We note that the resulting connectivity distributions scale as a power-law for a wide range of thresholds and the exponents only depend weakly on the choice of the threshold. The clustering coefficient of gene *i* is *C_i_* = (Σ_*k*>*j*≠*i*_ *A_ik_* *A_kj_* *A_ji_*)/[*k_i_*(*k_i_* −1)/2].

#### Web site

Interactive applications for the refinement of sets of homologous genes and the exploration of our modular decompositions of the expression data are available online. We also present details about the highly connected genes in each organism, the pairs of genes that are significantly correlated in two organisms, and the eight modules related to core processes in yeast (and their homologue modules before and after refinement) on our website at http://barkai-serv.weizmann.ac.il/ComparativeAnalysis.

## Supporting Information

Data S1 Testing the Robustness of Our Analyses with Respect to the Precise Values of Threshold Parameters This note includes **Figure S1** and **Figure S2**.
(38 KB PDF).

Data S2 Controls to Verify That Our Results Are Not Impaired by the Sparseness of the Available Expression Data This note includes **Figure S3**, **Figure S4**, and **Figure S5**.
(59 KB PDF).

Data S3 Testing for Coregulated Subsets within the Homologue Modules This note includes **Figure S6**.
(11 KB PDF).

Data S4 Comment on Missing Values in the Expression Data (3 KB PDF).

Data S5 Comment on the Size of the Human Dataset Used in This Work After this work was completed, we succeeded in processing the more than 2,000 human chip experiments deposited at the SMD. Removing genes and conditions with more than 90% missing values resulted in 1,474 expression profiles for 24,795 genes. Our Web tools (“GeneHopping” and “ModuleTree”) allow researchers to use also this updated dataset.
(3 KB PDF).

Figure S7 The Interactive Web Tool (137 KB PDF).

Figure S8 Statistical Analysis Comparing the Pairwise Correlations between Genes in One Organism to the Correlations between Their Respective Homologues (16 KB PDF).

Figure S9 Pairwise Correlations of C. elegans Homologues to the Yeast Heat-Shock Module (15 KB PDF).

Figure S10 Correlations between the Genes of Eight Representative Yeast Modules and Their Homologue Modules, Purified Modules, and Refined Modules (33 KB PDF).

Figure S11 Pairwise Correlations between Eight Transcription Modules of Known Function in Yeast and Their Refined Homologue Modules in the Five Other Organisms (11 KB PDF).

Figure S12 Histograms Showing the Number of Modules of One Organism with a Given Fraction of Homologues in Another Organism (29 KB PDF).

Figure S13 Connectivity Distributions for the Six Organisms in Separate Plots (19 KB PDF).

## Abbreviation

ISA: iterative signature algorithm
