# Supplementary material for: Similarities and Differences in Genome-Wide Expression Data of Six Organisms
Source: PLoS Biol. 2003 Dec 15;2(1):e9. doi: 10.1371/journal.pbio.0020009 (PMC300882; doi:10.1371/journal.pbio.0020009)
Supplement: Data S1 — This note includes Figure S1 and Figure S2. (38 KB PDF). [file pbio.0020009.sd001.pdf]

Supplementary Note I:

Choice of parameters

Our analysis uses several threshold parameters and the results are reported for specific values of these parameters. We verified that the precise choice of parameter has only a minor effect on the results. The following parameters were considered:

1. *Definition of homologues:*

   We examined how the parameters used for identifying homologues alter the gene content of the 'refined modules'. We repeated the analysis described in Fig. 1a of the main text for different choices of the BLAST parameters. Specifically, we varied the lower bound on the alignment, t_{align}, and the upper bound on the E-value, t_E. As is shown in Suppl. Fig. 1. changing t_E by a few orders of magnitude does not alter the refined modules significantly. The choice of t_{align} = 40% is more crucial for our analysis: Reducing this threshold leads to the inclusion of genes that share only a conserved domain, but are not real homologues. Choosing t_{align} much larger than 40% often results in very small homologue modules that contain not enough information for a sucessful refinement.

   We also considered using only genes that have a very high probability to be true orthologues (e.g. genes that are each other's best reciprocal BLAST hit). While the resulting 'homologue modules' in general contains less 'false positives' a very stringent homology criterion also reduces the number of 'true positives'. We note that the central feature of the signature algorithm used for the refinement of the homologue modules is its capability to distinguish a set of N_c co-expressed genes from a large number of unrelated genes (up to ~ N_c^2). Therefore, the use of relatively lenient homology criteria that results in many "candidate homologues", in general leads to better results.

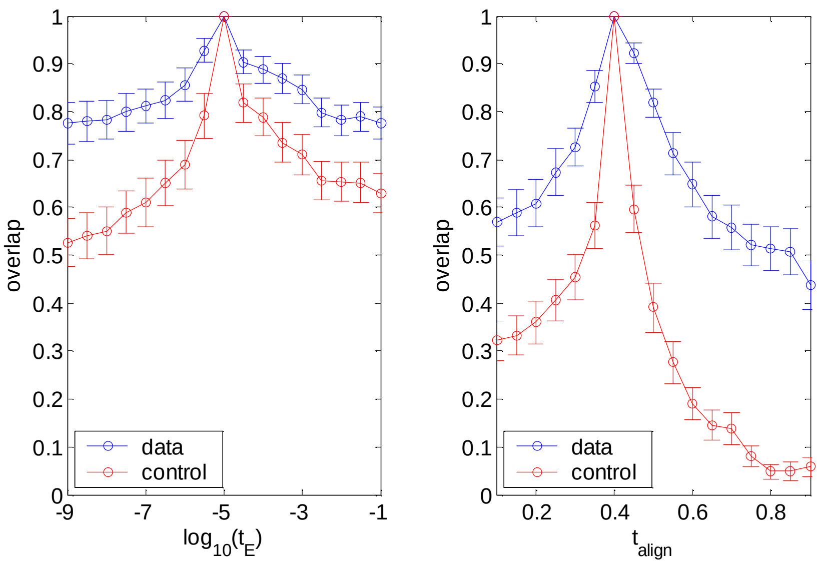

Supplementary Figure 1: Sensitivity of refined modules to changes in BLAST threshold parameters. We examined how the parameters used for identifying homologues alter the gene content of the 'refined modules'. We repeated the refinement procedure (c.f. main text and Figure 1a) for different choices of the BLAST parameters. Specifically, we varied the upper bound on the E-value, t_E (left panel) and the lower bound on the alignment, t_{align} (right panel). For each threshold value we computed the overlaps between the resulting modules and those obtained for our default value ($t_E = 10^{-5}$ and t_{align} = 40%). (The overlap is defined as the ratio between the size of the intersect and the union of the respective sets of genes.) The blue circles indicate the average overlap of the eight refined modules in the five organisms. We also show a control

obtained by using sets of randomly selected genes that have the same size as the homologue modules as input to the refinement procedure (red circles).

2. *Correlation threshold for defining edges in the expression networks:*

Two genes were connected by an edge if the Pearson correlation between their expression profiles exceeds a certain threshold. Due to the very different sizes of the respective sets of expression data, we demanded that the average connectivity <*k*> (rather than the minimal correlation) is identical in all expression networks and fixed it to <*k*> = 0.001. As is shown in Suppl. Fig. 2, the connectivity distribution follows a power-law for a wide range of <*k*>. The values for the powers slightly increase for smaller values of <*k*>.

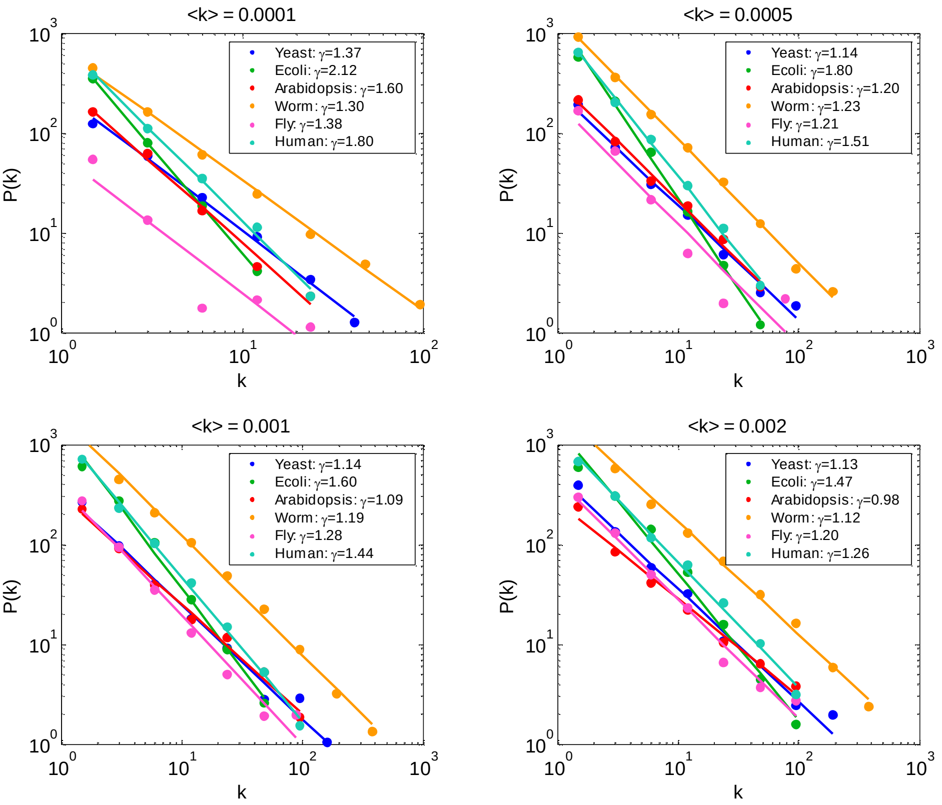

Supplementary Figure 2: Sensitivity of connectivity distributions to choice of threshold on correlation. We checked how different thresholds for the Pearson correlations used to define the edges of the expression networks affect their connectivity distributions. We demanded that the average connectivity <*k*> (rather than the minimal correlation) is identical in all expression networks and fixed it to the values indicated above each plot. Note that the all distributions follow a power-law. The powers slightly increase for smaller values of <*k*>.
