# Supplementary material for: Similarities and Differences in Genome-Wide Expression Data of Six Organisms
Source: PLoS Biol. 2003 Dec 15;2(1):e9. doi: 10.1371/journal.pbio.0020009 (PMC300882; doi:10.1371/journal.pbio.0020009)
Supplement: Data S2 — This note includes Figure S3, Figure S4, and Figure S5. (59 KB PDF). [file pbio.0020009.sd002.pdf]

Supplementary Note II:

Sparseness of the data

For most organisms the available expression are still quite sparse and contain very different conditions. We performed the following controls to verify that our results are not impaired by these limitations:

1. *Module refinement:*

   To examine how the choice of experimental conditions affects the correlation between the eight representative "homologue modules", we repeated the refinement procedure described in main text 100 times, each time using only a fraction of randomly selected expression profiles. We then measured the mean and standard deviation for the "overlap" between the resulting modules and those we obtained for the full dataset. (The overlap is defined as the ratio between the size of the intersection and the union of the respective sets of genes.) The results are summarized in Suppl. Fig. 3. We find that typically, the removal of a subset of conditions does not significantly change the gene content of the refined modules. However, at the quantitative level there are differences both with respect to the organisms and the modules:

   - The yeast modules are by far the most robust. This is due to the large number of expression profiles available for *S. cerevisiae*. It may also reflect the quality of the data, the important role of transcriptional regulation in yeast as well as fact that no sequenced-based gene mapping is required in this case.
   - Not surprisingly, the size of the dataset affects the robustness of the modules. Thus, even when ignoring half of the 547 expression profiles for *C. elegans*, on average the resulting modules are still quite similar to the original ones (~70% overlap). In contrast, for the smaller datasets (*E. coli* and *D. melonogaster*) the overlap decreases much more rapidly upon reducing the fraction of conditions used. An interesting observation is that also the overlap profiles for the human expression data behave similarly, although our human dataset contains about twice as many conditions. This is likely to reflect that the human data are noisier.
   - We also observed differences in robustness between the modules: The gene contents of the ribosomal protein modules (MRP for *E. coli*) are the least affected by the removal of expression profiles, reflecting their strong co-regulation under many experimental conditions.

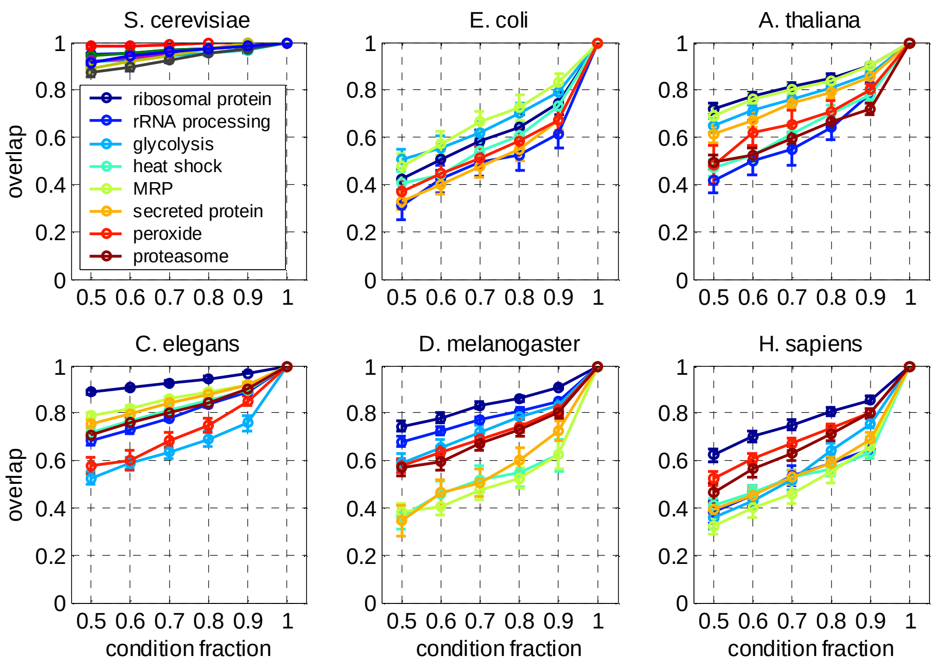

Supplementary Figure 3: Sensitivity of refined modules to reduction of conditions in dataset. We repeated the refinement procedure (c.f. main text and Figure 1a) 100 times, each time using only a fraction of randomly selected expression profiles. We then measured the mean and standard deviation for the overlap between the resulting modules and those we obtained for the full dataset. (The overlap is defined as the ratio between the size of the intersection and the union of the respective sets of genes.) The results the eight representative modules (legend) are summarized for each organism.

2. *Module correlations:*

Secondly, we investigated how a reduction of the number of expression profiles affects our statements about the regulatory relations between the refined modules in each organism. To this end we repeated our analysis using the reduced datasets. We reevaluated the correlations between the sets of conditions and computed their mean and standard deviation as a function of the fraction of removed conditions. We find that in general the regulatory relations are very insensitive to the subset of conditions used (Suppl. Fig. 4). For the largest datasets (yeast and *C. elegans*) the standard deviations of the correlation coefficients do not exceed 0.1, even when removing half of the expression profiles. Also for the other organisms most correlations fluctuate by less than 0.2 when using only 50% of the data. In particular, the three biologically interesting relations that we mention in the text are very robust (Suppl. Fig. 5): The ribosomal protein (RP) - heat shock correlations are negative within one standard deviation only for yeast and *Drosophila*. Furthermore, the statements about the correlations with common signs for all organisms remain valid when considering only subsets of the expression data.

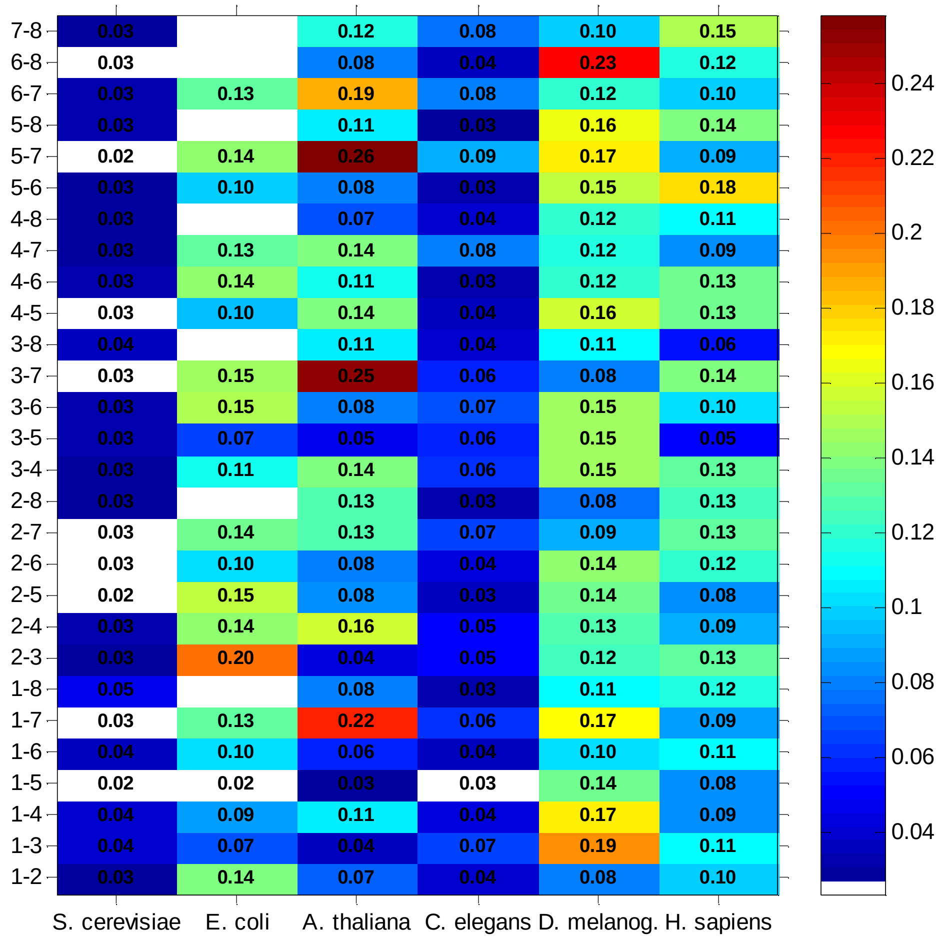

Supplementary Figure 4: Sensitivity of correlations between refined modules to reduction of experimental conditions in each dataset. We repeated the refinement procedure (c.f. main text and Fig. 1a) 100 times, each time removing 50% of the available expression profiles at random. We reevaluated the correlations between the sets of conditions and computed their standard deviation (numbers shown) for all organisms. (c.f. Fig. 2a for module names).

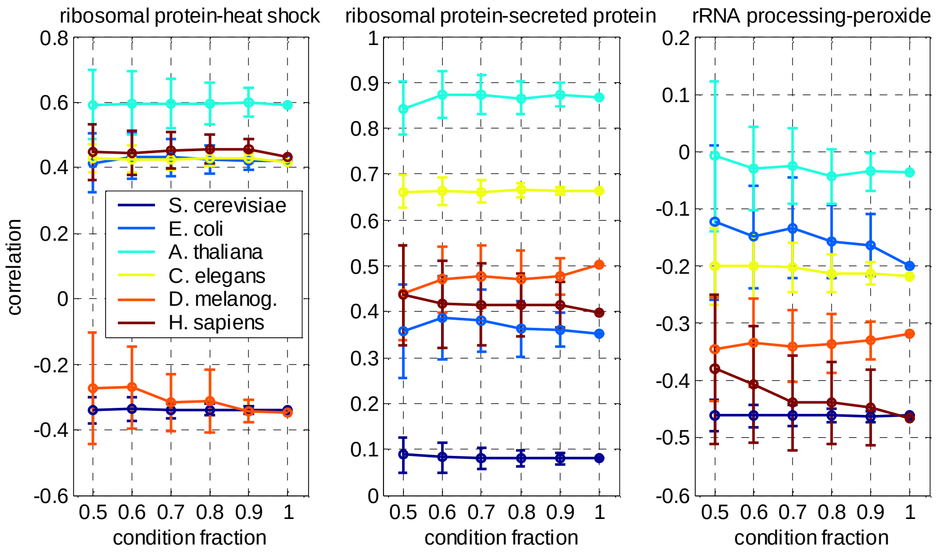

Supplementary Figure 5: Sensitivity to reduction of experimental conditions for three correlations between refined modules. We repeated the refinement procedure (c.f. main text and Fig. 1a) 100 times, each time removing a fraction of the available expression profiles at random. We reevaluated the correlations between the sets of conditions and computed their mean and standard deviation. The results for three correlations between modules are shown for the six organisms (legend).

Our results indicate that the eight representative modules and most of their correlations are unlikely to change significantly when new expression data becomes available. The experience with our constantly growing yeast expression database is that the most fundamental transcription modules as well as their correlations could already be established reliably when only a few hundred expression profiles were available. Yet, the identification of more specific subsets of co-regulated genes obviously requires a sufficient number of experiments that resolve specific responses. Thus, we expect that the resolution of the modular decomposition of the various transcription programs will increase when more expression data are accumulated. Evidently, designing innovative experiments that force the organisms into so far unknown transcriptional responses are required to uncover new transcription modules.
