# Supplementary material for: Similarities and Differences in Genome-Wide Expression Data of Six Organisms
Source: PLoS Biol. 2003 Dec 15;2(1):e9. doi: 10.1371/journal.pbio.0020009 (PMC300882; doi:10.1371/journal.pbio.0020009)
Supplement: Data S3 — This note includes Figure S6. (11 KB PDF). [file pbio.0020009.sd003.pdf]

Supplementary Note III:

Substructures in the homologue modules

In general a homologue module may contain more than one group of co-regulated genes. Only if one group is dominant the purification procedure will filter out this set of genes. Indeed we were able to identify substructures within several purified modules by clustering all pairwise gene-correlations (results for all modules are available on our website): For example, the glycolysis module in *C. elegans* consists of 16 genes that can be separated into two clusters of identical size (Suppl. Fig. 6). This could indicate that (part of) the glycolysis pathway is mediated by different sets of isozymes (which evolved from common ancestors), each only induced under specific conditions or in a distinct developmental stage. In fact we observed that the refinement of such modules is more sensitive to changes in BLAST parameters, because adding only a few homologues associated with one sub-module can easily bias the regulatory context. Our interactive "Gene-Hopping" tool (Suppl. Fig. 7) can be used for a detailed study of this point for any set of genes.

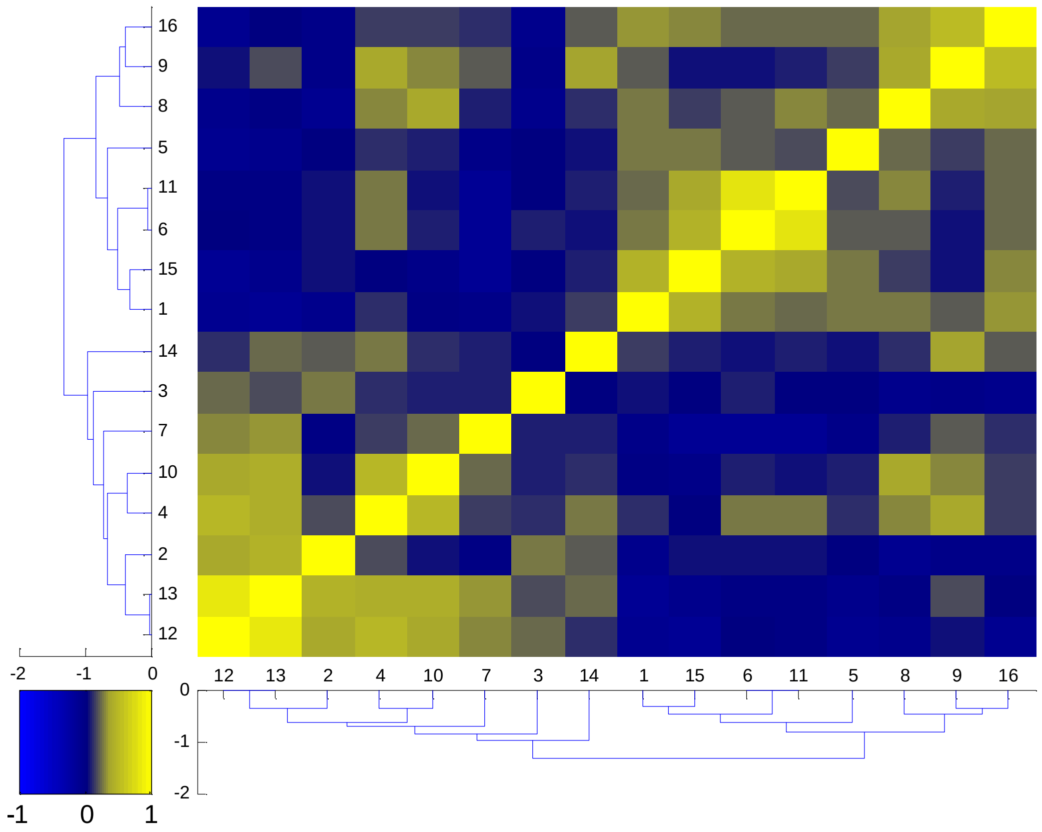

Supplementary Figure 6: Substructures in the homologue modules. Homologue modules may contain more than one group of co-regulated genes. We clustered all pairwise gene-correlations to identify substructures within the modules. The figure shows the results for the glycolysis module in *C. elegans* consisting of 16 genes that can be separated into two clusters of identical size. Results for all modules are available on our website.
