# Supplementary material for: Similarities and Differences in Genome-Wide Expression Data of Six Organisms
Source: PLoS Biol. 2003 Dec 15;2(1):e9. doi: 10.1371/journal.pbio.0020009 (PMC300882; doi:10.1371/journal.pbio.0020009)
Supplement: Data S4 — (3 KB PDF). [file pbio.0020009.sd004.pdf]

Supplementary Note IV:

Missing data points

Missing data points present a serious challenge in the analysis of expression data. We investigated several techniques to deal with such points, including rather sophisticated algorithms to impute their values based on the existing data (Troyanskaya et al, Bioinformatics 2001; 17(6):520-5). We decided that the most conservative approach is to put these values to zero in the normalized matrices of the expression log-ratios. Thus, these points do not contribute to the scores in the signature algorithm. However, expression profiles that include only a very small fraction of the genes, as well as genes whose expression was recorded only under few experimental conditions cannot be associated reliably to transcription modules and should therefore be excluded from the analysis.
