# Supplementary material for: Similarities and Differences in Genome-Wide Expression Data of Six Organisms
Source: PLoS Biol. 2003 Dec 15;2(1):e9. doi: 10.1371/journal.pbio.0020009 (PMC300882; doi:10.1371/journal.pbio.0020009)
Supplement: Data S5 — After this work was completed, we succeeded in processing the more than 2,000 human chip experiments deposited at the SMD. Removing genes and conditions with more than 90% missing values resulted in 1,474 expression profiles for 24,795 genes. Our Web tools (“GeneHopping” and “ModuleTree”) allow researchers to use also this updated dataset. (3 KB PDF). [file pbio.0020009.sd005.pdf]

Supplementary Note V:

Note on Human dataset

The presence of more than 60.000 ESTs makes the handling of the human expression data the most difficult. In order to perform the normalization of the expression data in our Matlab system we had to restrict the size of the human dataset. However, this restriction is in no way a limitation of the analysis method itself. In fact, as we pointed out previously (Bergmann et al., Phys. Rev. E 67, 031902), the (iterative) signature algorithm is especially efficient for the analysis of large-scale data. We are currently considering the use of a different computer platform to attempt the analysis of all available expression profiles.
