# Supplementary material for: Similarities and Differences in Genome-Wide Expression Data of Six Organisms
Source: PLoS Biol. 2003 Dec 15;2(1):e9. doi: 10.1371/journal.pbio.0020009 (PMC300882; doi:10.1371/journal.pbio.0020009)
Supplement: Figure S7 — (137 KB PDF). [file pbio.0020009.sg001.pdf]

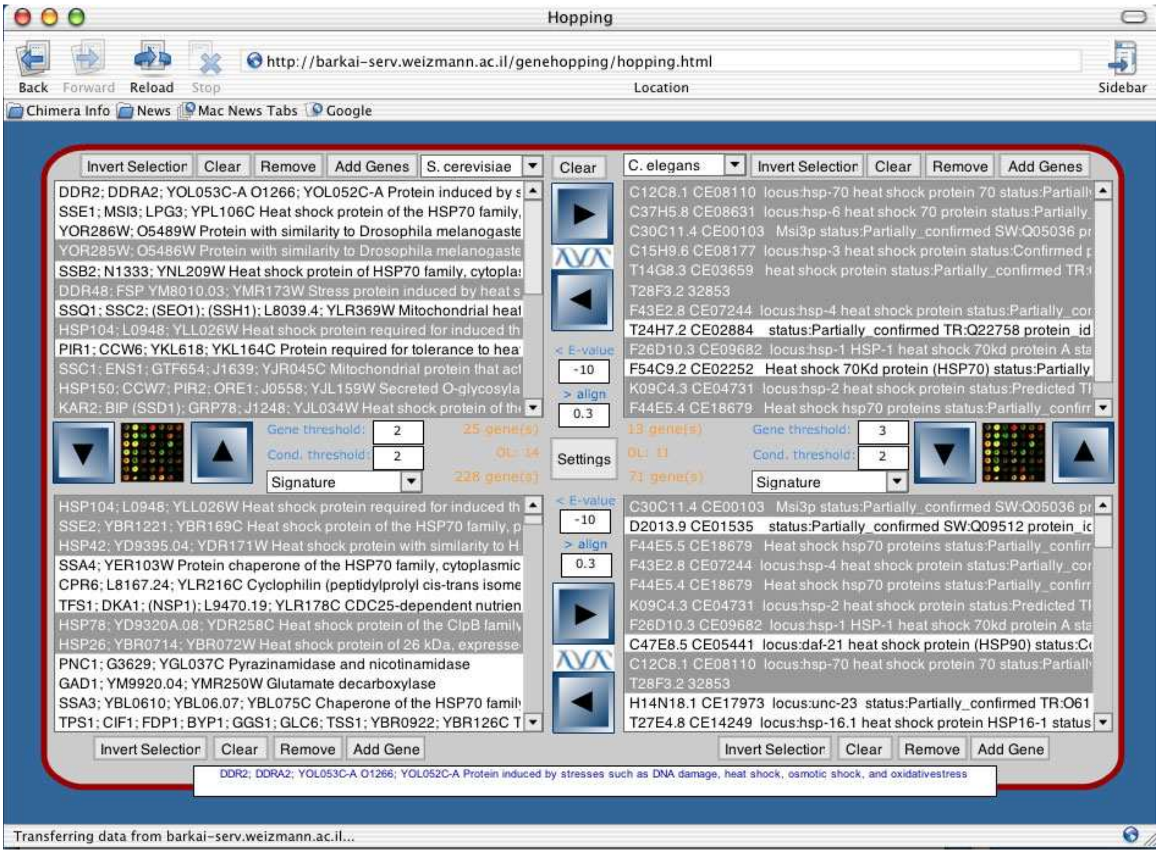

Supplementary Figure 7: Interactive web tool for integrated exploration of our database. Investigators can assemble gene sets of their interest. Such sets can be mapped (horizontally) to other organisms according to sequence similarity. Alternatively, co-regulated genes can be extracted and refined according to the expression data of one organism (vertical mapping). The parameters determining stringency in sequence similarity (i.e. *E*-value and alignment-fraction) as well as co-regulation (gene- and condition threshold) can be tuned interactively. This online application is available on our website.
