# Supplementary material for: Similarities and Differences in Genome-Wide Expression Data of Six Organisms
Source: PLoS Biol. 2003 Dec 15;2(1):e9. doi: 10.1371/journal.pbio.0020009 (PMC300882; doi:10.1371/journal.pbio.0020009)
Supplement: Figure S8 — (16 KB PDF). [file pbio.0020009.sg002.pdf]

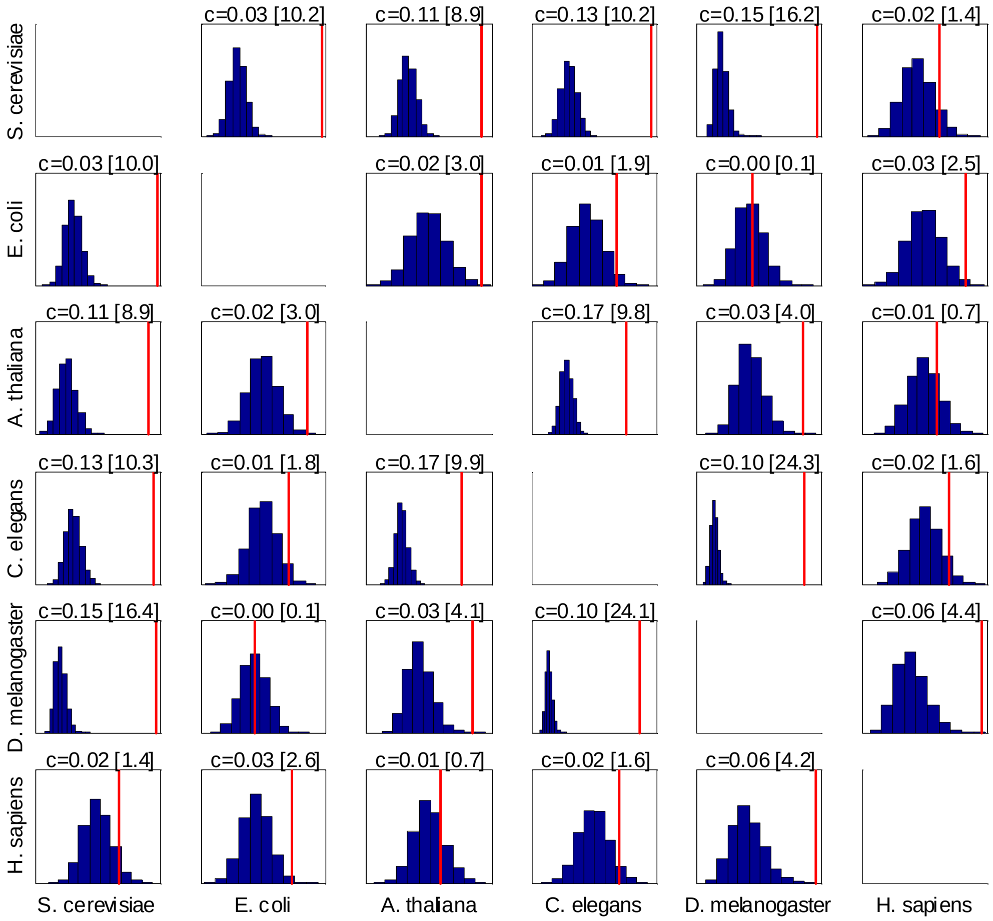

Supplementary Figure 8: Statistical analysis comparing the pair-wise correlations $C_{ij}^{(ref)}$ between all genes in the reference organism (indicated on the left) to the pair-wise correlations $C_{ij}^{(homol)}$ of the respective homologues in a second organism (bottom). The indices i and j go over all genes whose sequence could be aligned along 80% of the sequence by the BLAST algorithm (50% for *E. coli*). C_{ij} refer to the Pearson correlations between the expression profiles of the respective organism. The correlation coefficient between the pair-wise correlations $C_{ij}^{(ref)}$ and $C_{ij}^{(hom)}$ is given by $c = \sum_{i<j} \hat{C}_{ij}^{(ref)} \cdot \hat{C}_{ij}^{(\mathrm{hom})}$, where $\hat{C}_{ij} = \dfrac{C_{ij} - \langle C_{ij} \rangle}{\sqrt{\sum_{i<j} (C_{ij} - \langle C_{ij} \rangle)^2}}$. c is indicated by a red line in each plot and shown in brackets. For comparison we computed c_{rand} for 5,000 controls obtained by re-shuffling the genes of the reference organism at random. We show the normalized distribution for c_{rand} in blue. In most cases c is several standard deviations above the mean of the control distribution (c.f. *Z*-scores in square brackets). Human only correlates significantly with the fruit-fly while no correlation is observed between *E. coli* and *D. melanogaster*. For details about the pairs of genes that are co-expressed in two organisms see Suppl. Online Material.
