# Supplementary material for: Similarities and Differences in Genome-Wide Expression Data of Six Organisms
Source: PLoS Biol. 2003 Dec 15;2(1):e9. doi: 10.1371/journal.pbio.0020009 (PMC300882; doi:10.1371/journal.pbio.0020009)
Supplement: Figure S9 — (15 KB PDF). [file pbio.0020009.sg003.pdf]

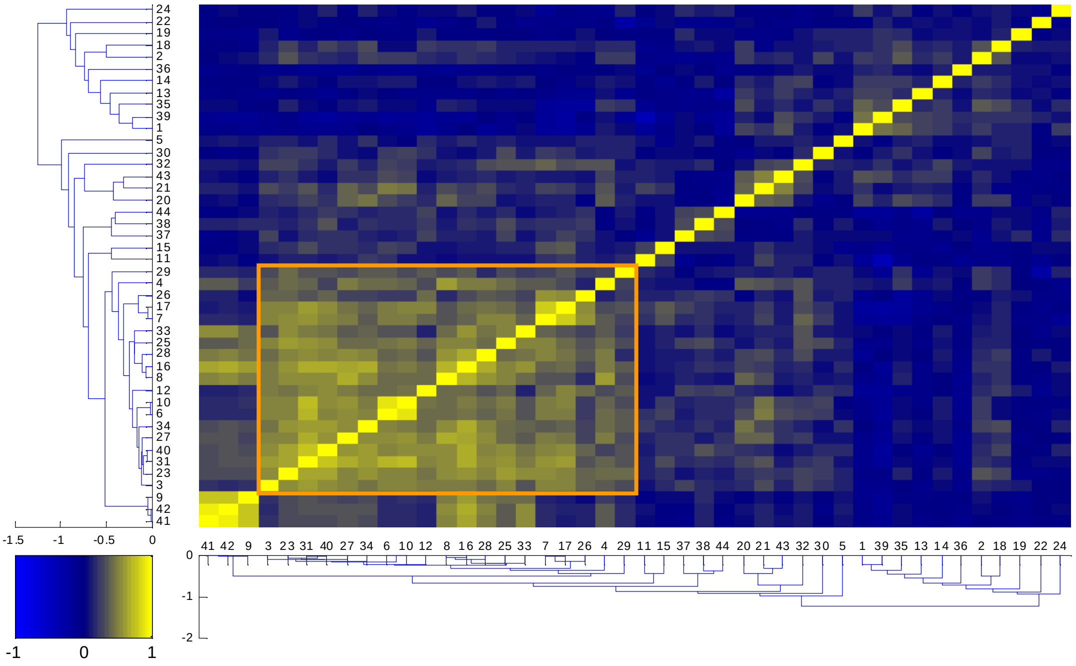

Supplementary Figure 9: Pair-wise correlations of *C. elegans* homologues to the yeast heat shock module. The Pearson correlations between the corresponding expression profiles are indicated by color code (see color-bar in the lower left). Only a subset of the homologues is strongly correlated (orange box). The expression profiles of the other homologues are less coherent, indicating that the corresponding genes are not co-regulated with this subset.
