# Supplementary material for: Similarities and Differences in Genome-Wide Expression Data of Six Organisms
Source: PLoS Biol. 2003 Dec 15;2(1):e9. doi: 10.1371/journal.pbio.0020009 (PMC300882; doi:10.1371/journal.pbio.0020009)
Supplement: Figure S10 — (33 KB PDF). [file pbio.0020009.sg004.pdf]

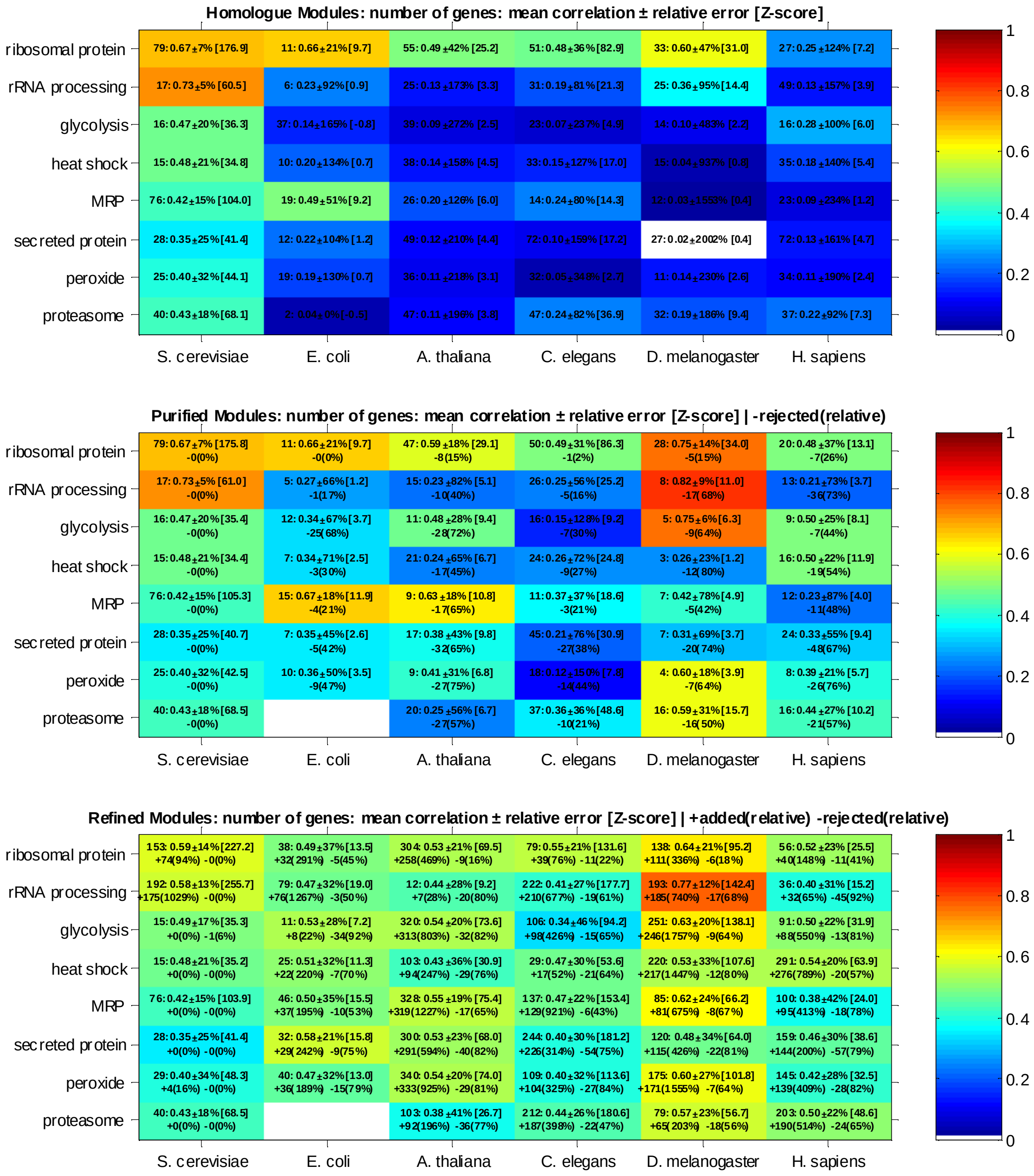

Supplementary Figure 10: Correlations between the genes of eight representative yeast modules (left) and their homologues in other organisms (top panel). The colors indicate the average pair-wise correlation (see colorbar), which is shown together with the number of genes as well as the standard deviation. We considered 10,000 random modules of the same size in order to estimate the *Z*-scores for the observed correlation (indicated in square brackets). We used the signature algorithm to reject genes that are not correlated with the bulk of the homologues and add other genes that are correlated to these ‘purified’ homologues. The corresponding correlations are indicated in the middle and bottom panels. Details about the genes are available in the interactive version of this figure in the Suppl. Online Material.
