# Supplementary material for: Similarities and Differences in Genome-Wide Expression Data of Six Organisms
Source: PLoS Biol. 2003 Dec 15;2(1):e9. doi: 10.1371/journal.pbio.0020009 (PMC300882; doi:10.1371/journal.pbio.0020009)
Supplement: Figure S11 — (11 KB PDF). [file pbio.0020009.sg005.pdf]

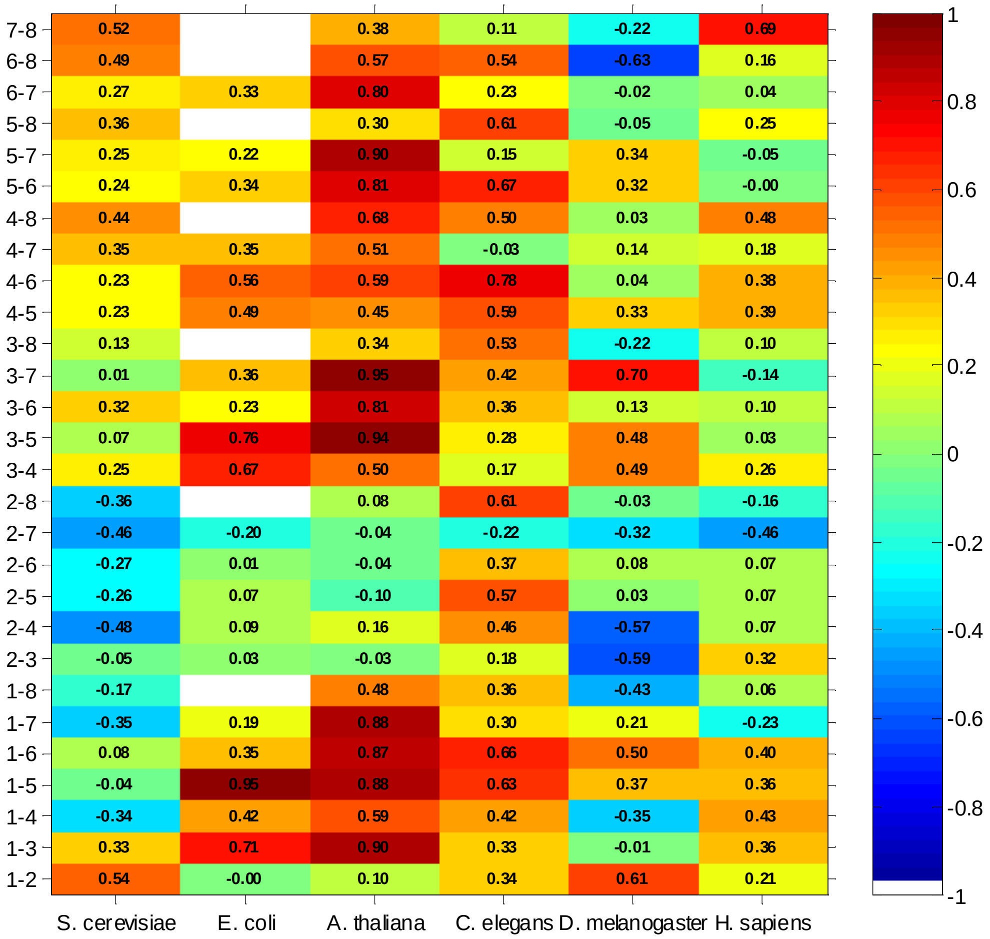

Supplementary Figure 11: The table shows all pair-wise correlations between eight transcription modules of known function in yeast, and their (refined) homologue modules in the five remaining organisms (c.f. Fig. 2). Specifically, we computed the correlation coefficients between the "condition profiles" generated by the signature algorithm for each module based on the respective expression data (Methods).
