# Supplementary material for: Similarities and Differences in Genome-Wide Expression Data of Six Organisms
Source: PLoS Biol. 2003 Dec 15;2(1):e9. doi: 10.1371/journal.pbio.0020009 (PMC300882; doi:10.1371/journal.pbio.0020009)
Supplement: Figure S12 — (29 KB PDF). [file pbio.0020009.sg006.pdf]

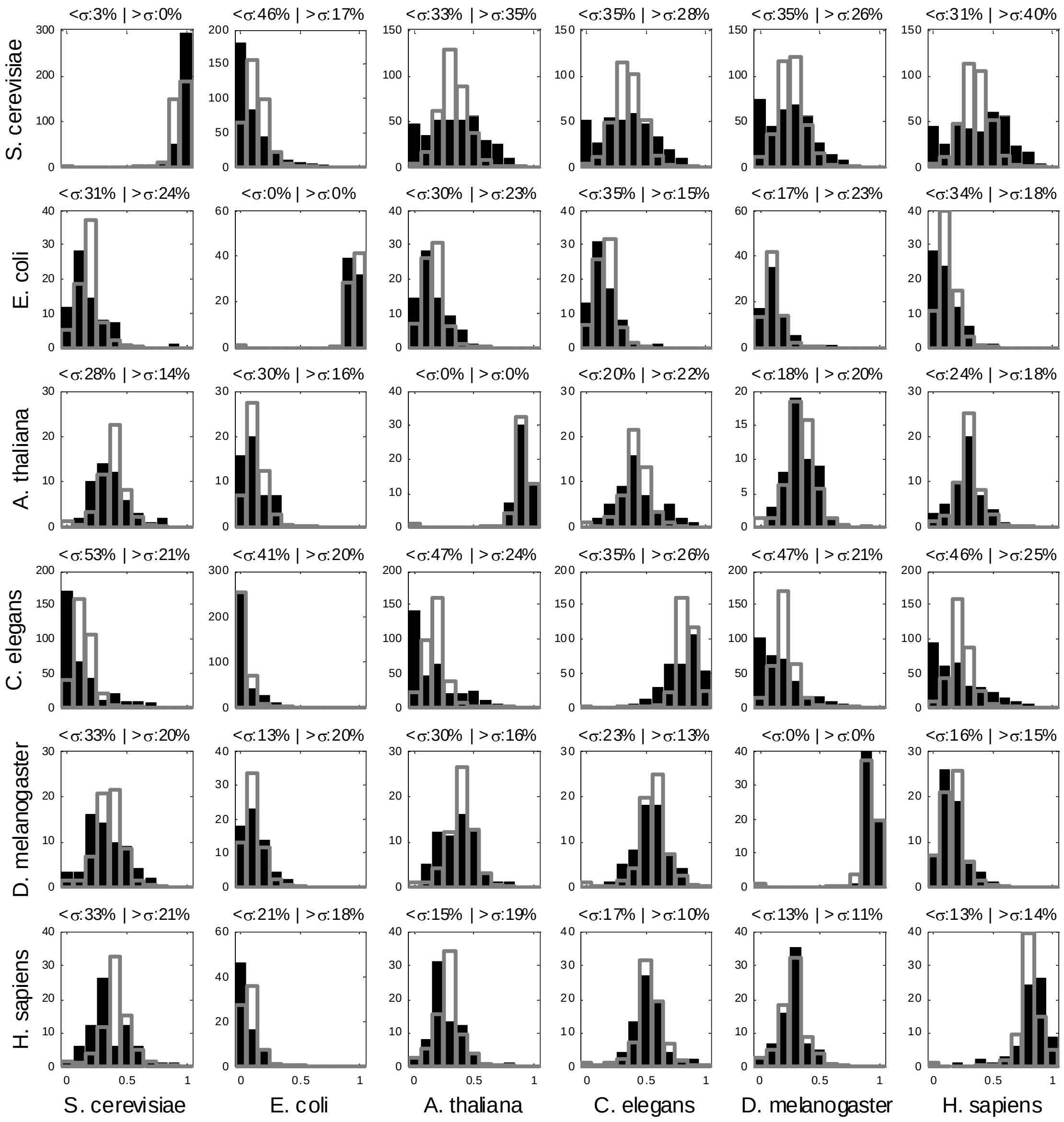

Supplementary Figure 12: Histograms (black bars) for the number of modules of the organism indicated on the left with a given fraction of homologues in another organism (bottom). The fraction is given by the number of module genes that possess at least one homologue divided by the size of the module. For comparison we computed the distributions for 5,000 controls using modules of the same size, but with randomly selected genes (gray). The observed distributions usually deviate significantly from the control. In particular, the number of modules with very few homologues (less than one-sigma [< σ] of the control distribution) is more than expected. Moreover, for yeast (and to less extend for *C. elegans*) also the number of modules with very many homologues (more than one-sigma [> σ] of the control distribution) is significant.
