# Supplementary material for: Similarities and Differences in Genome-Wide Expression Data of Six Organisms
Source: PLoS Biol. 2003 Dec 15;2(1):e9. doi: 10.1371/journal.pbio.0020009 (PMC300882; doi:10.1371/journal.pbio.0020009)
Supplement: Figure S13 — (19 KB PDF). [file pbio.0020009.sg007.pdf]

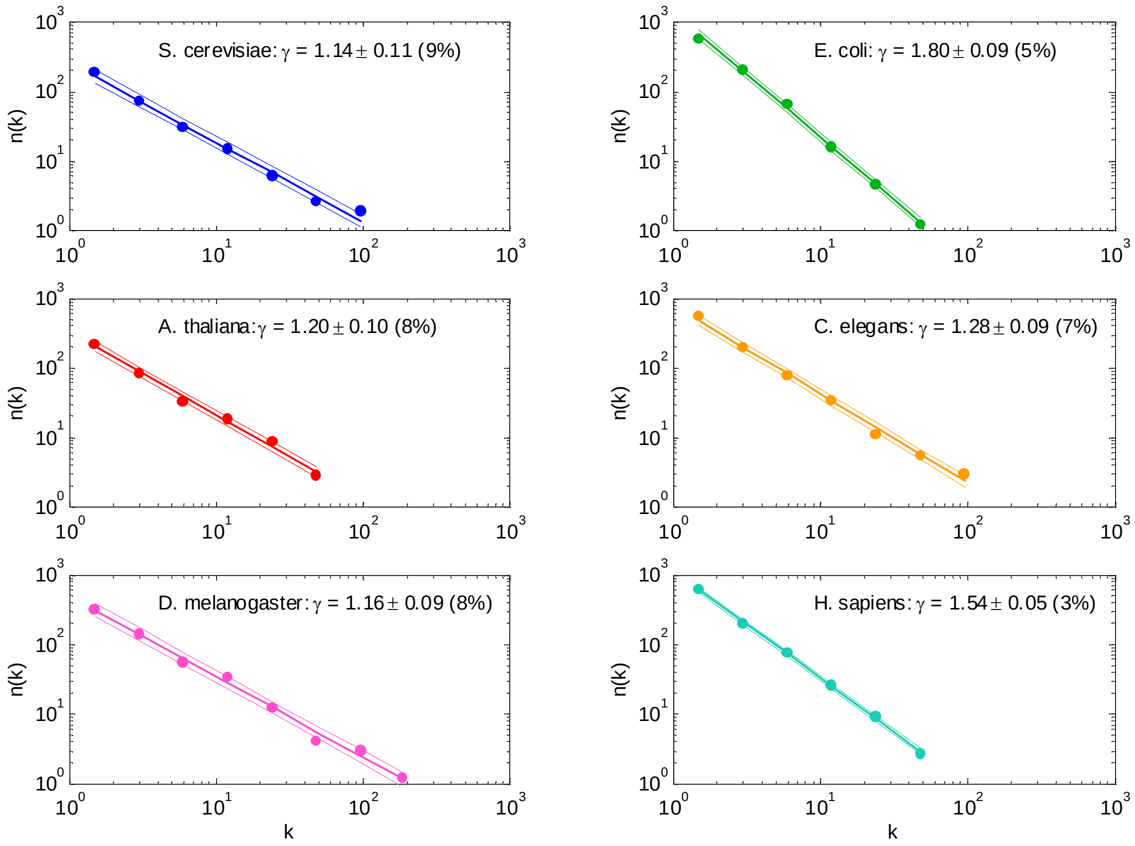

Supplementary Figure 13: Connectivity distributions: The number of genes n(k) with connectivity k is plotted against k using logarithmic binning (Methods). The thick lines indicate the best linear fit to the log values of the bin centers and the normalized counts. The thin lines correspond to the one-sigma confidence interval. For each of the six organisms n(k) is distributed as a power-law, $n(k) \sim k^{-\gamma}$, with similar exponents as indicated in each plot. The error corresponds to the maximal deviation within the shown confidence interval.
